# P2Y_12_-dependent activation of hematopoietic stem and progenitor cells promotes emergency hematopoiesis after myocardial infarction

**DOI:** 10.1007/s00395-022-00927-6

**Published:** 2022-03-30

**Authors:** Hana Seung, Jan Wrobel, Carolin Wadle, Timon Bühler, Diana Chiang, Jasmin Rettkowski, Nina Cabezas-Wallscheid, Béatrice Hechler, Peter Stachon, Alexander Maier, Christian Weber, Dennis Wolf, Daniel Duerschmied, Marco Idzko, Christoph Bode, Constantin von zur Mühlen, Ingo Hilgendorf, Timo Heidt

**Affiliations:** 1 Department of Cardiology and Angiology I, University Heart Center Freiburg – Bad Krozingen, Medical Faculty, University of Freiburg, Hugstetterstr. 55, 79106 Freiburg, Germany; 2 Spemann Graduate School of Biology and Medicine (SGBM), University of Freiburg, Freiburg, Germany; 3 Faculty of Biology, University of Freiburg, Freiburg, Germany; 4 Max-Planck Institute for Immunobiology and Epigenetics, Freiburg, Germany; 5 Fédération de Médecine Translationnelle de Strasbourg (FMTS), Université de Strasbourg, INSERM, BPPS UMR_S 1255, Etablissement Français du Sang (EFS)-Grand Est, 67000 Strasbourg, France; 6 Department of Cardiology, Angiology, Haemostaseology and Medical Intensive Care, Medical Faculty Mannheim, University Medical Centre Mannheim, Heidelberg University, Mannheim, Germany; 7 European Center for AngioScience (ECAS) and German Center for Cardiovascular Research (DZHK) Partner Site Heidelberg/Mannheim, Mannheim, Germany; 8 Department of Internal Medicine II, Division of Pulmonology, Medical University of Vienna, Vienna, Austria

**Keywords:** Myocardial infarction, Inflammation, Hematopoiesis, ADP, P2Y_12_ receptor

## Abstract

Emergency hematopoiesis is the driving force of the inflammatory response to myocardial infarction (MI). Increased proliferation of hematopoietic stem and progenitor cells (LSK) after MI enhances cell production in the bone marrow (BM) and replenishes leukocyte supply for local cell recruitment to the infarct. Decoding the regulation of the inflammatory cascade after MI may provide new avenues to improve post-MI remodeling. In this study, we describe the influence of adenosine diphosphate (ADP)-dependent P2Y_12_-mediated signaling on emergency hematopoiesis and cardiac remodeling after MI. Permanent coronary ligation was performed to induce MI in a murine model. BM activation, inflammatory cell composition and cardiac function were assessed using global and platelet-specific gene knockout and pharmacological inhibition models for P2Y_12_. Complementary in vitro studies allowed for investigation of ADP-dependent effects on LSK cells. We found that ADP acts as a danger signal for the hematopoietic BM and fosters emergency hematopoiesis by promoting Akt phosphorylation and cell cycle progression. We were able to detect P2Y_12_ in LSK, implicating a direct effect of ADP on LSK via P2Y_12_ signaling. P2Y_12_ knockout and P2Y_12_ inhibitor treatment with prasugrel reduced emergency hematopoiesis and the excessive inflammatory response to MI, translating to lower numbers of downstream progeny and inflammatory cells in the blood and infarct. Ultimately, P2Y_12_ inhibition preserved cardiac function and reduced chronic adverse cardiac remodeling after MI. P2Y_12_-dependent signaling is involved in emergency hematopoiesis after MI and fuels post-ischemic inflammation, proposing a novel, non-canonical value for P2Y_12_ antagonists beyond inhibition of platelet-mediated atherothrombosis.

**Supplementary Information:**

The online version contains supplementary material available at 10.1007/s00395-022-00927-6.

## Introduction

Inflammation is an essential prerequisite for wound healing and cardiac remodeling after myocardial infarction (MI) [43]. Acute ischemic myocardial injury triggers a systemic inflammatory response including innate immune cell production and release, local recruitment, maturation and apoptosis [19, 36, 50]. The infiltrating immune cells orchestrate breakdown and removal of necrotic debris as well as lesion repair with collagen deposition and neovascularization [44, 78]. These changes arise in the necrotic area but also stretch to the borders of the infarcted myocardium and even remote zones [34]. However, excessive inflammation or inadequate resolution of the inflammatory response after MI may advocate adverse cardiac remodeling and accelerate heart failure [47, 58].

Injured cardiac cells secrete various chemoattractants that have been appreciated to locally regulate post-MI inflammation [5, 15]. In addition, activated platelets release a plethora of prothrombotic factors and immunoregulatory cytokines to promote endothelial activation and facilitate platelet-leukocyte-complex formation [9, 22, 70]. Following the resulting cytokine gradients, immune cells are recruited to the site of injury [5]. As a consequence of the high leukocyte turnover after MI, reservoirs of preformed innate immune cells in the bone marrow (BM), spleen and blood are rapidly depleted and rely on immediate resupply by emergency hematopoiesis to compensate for the excessive demand [36, 49].

Upstream in the hematopoietic hierarchy, hematopoietic stem and progenitor cells, defined as LSK cells based on their characteristic surface expression pattern (Lineage ^neg^, Sca-1^+^, c-Kit^+^) [42], are activated and enter the cell cycle to increase the production of myeloid lineage progeny, predominately [11]. Multiple cascades have previously been described that transport information from the site of injury to the BM [65], involving blood borne factors, e.g. interleukin-1β (IL-1β) and interferons [3, 12, 57] or extravascular sympathetic nervous signaling [6, 10, 25]. IL-1β was shown to activate emergency hematopoiesis both directly by acting on LSK and indirectly by downregulating retention factors in the hematopoietic BM niche that are necessary for HSC homeostasis [57]. Likewise, noradrenaline released by sympathetic nerve fibers decreases the retention factor CXCL12 in the BM niche, subsequently elevating LSK proliferation [25].

Importantly, extracellular nucleotides, e.g. adenosine 5′-triphosphate (ATP), adenosine 5′-diphosphate (ADP) and uridine-5′-diphosphate (UDP), represent another subset of soluble danger signals after myocardial injury that activate purinergic receptors [24, 29, 46]. In cardiovascular disease, ADP is a key regulator of platelet activity and inhibition of the purinergic receptor P2Y_12_ has thus emerged as an important therapeutic strategy to reduce recurrent cardiovascular events [72, 75].

Intriguingly, LSK cells have been reported to also express purinergic receptors [13, 56]. While purinergic P2X receptors on hematopoietic progenitors have recently been described to impact cell trafficking, the role of ADP-sensitive P2Y receptors has not yet been characterized [1].

In this study, we show that ADP acts as danger signal for the hematopoietic BM after MI and fosters emergency hematopoiesis by promoting Akt phosphorylation and cell cycle progression in LSK via P2Y_12_-dependent signaling. Using platelet-specific as well as global P2Y_12_-deficiency models and treatment with the potent P2Y_12_ inhibitor prasugrel, we demonstrate that P2Y_12_ inhibition reduces emergency hematopoiesis and the excessive inflammatory response to MI, subsequently preserving cardiac function and preventing adverse cardiac remodeling after MI. Targeting the ADP-dependent, P2Y_12_ receptor-mediated signaling pathway after MI may thus exert beneficial, non-canonical effects beyond inhibition of platelet activation.

## Methods

### Experimental animals

We used female C57BL/6 (WT), C57BL/6-P2Y_12_-deficient (global P2Y_12_-KO) and C57BL/6-Tg (UBC-GFP) 30Scha/J mice aged 8–13 weeks (Charles River, Janvier) in our study. Age-matched mice were randomly assigned to treatment or control groups. The study was conducted according to GV-SOLAS guidelines and approved by the local ethics committee.

### Bone marrow transplantation

Recipient C57BL/6-Tg (UBC-GFP) 30Scha/J mice were lethally irradiated with a cumulative dose of 9.5 Gy. 6 h after irradiation, bone marrow was reconstituted with 5 × 10^6^ full bone marrow cells of B6.PF4^cre/wt^:P2Y_12_^fl/fl^ animals via tail vein [52]. Animals received antibiotic treatment with sulfadimidine 1 mg/ml and trimethoprim 0.2 mg/ml for 4 weeks after transplantation and bone marrow was given 16 weeks to engraft and return to homeostasis prior to further experiments. As a result, bone marrow of reconstituted UBC-GFP mice was completely replaced by bone marrow of platelet-specific P2Y_12_-deficient mice. Hence, only platelets and megakaryocytes of these mice lack the P2Y_12_ receptor while P2Y_12_ is still present in all other cell types. These chimeras will be called P2Y_12_(plt)^−/−^GFP chimera throughout the manuscript.

### Prasugrel and ASA treatment

Treatment was induced one day prior to MI with an initial loading dose and continued once daily after MI. Using a gavage feeding needle, prasugrel (5 mg/kg body weight) [60] or acetylsalicylic acid (ASA) (10 mg/kg body weight) was applied in the treatment group, whereas the control group received the vehicle solution.

### Myocardial infarction surgery

Anesthesia was induced by intraperitoneal injection (i.p.) of 100 mg/kg ketamine (Zoetis) and 10 mg/kg xylazine (Bayer Vital). Analgesia was initiated approximately 30 min before surgery by subcutaneous (s.c.) injection of 0.1 mg/kg buprenorphine. To compensate for perioperative dehydration due to blood loss and perspiration, 20 ml/kg isotonic 5% glucose solution (B. Braun) in 0.9% NaCl (9 mg/ml) was applied i.p.. Ventilation was set to a positive end-inspiratory pressure (PEEP) of 5 mbar, a respiratory rate of 110/min and an inspiration/expiration ratio of 1/1.5 with a small animal respirator (TSE Systems). Oxygen saturation, heart rate, and respiratory rate were monitored throughout the procedure by a MouseOX system (Starr Life Sciences). Anesthesia was maintained by addition of 0.5–2% isoflurane (AbbVie) during surgery. After right lateral positioning of the animal and skin disinfection, left lateral thoracotomy was performed between the 3rd and 4th rib. Opening of the pericardium allowed for identification of the left anterior descending (LAD) coronary artery. Permanent LAD ligation was performed with one single suture in the proximal middle third of the LAD, using 8-0 prolene suture (Ethicon). After evacuating the pneumothorax, chest and skin wounds were closed using a 5-0 prolene suture (Ethicon).

### Light transmission aggregometry

600 µl venous blood was acquired by tail vein puncture and added to 400 µl enoxaparin (0.3 mg/ml). Samples were spun down twice at 100 G for 5 min each at room temperature. The supernatant retrieved from the sample created platelet-rich plasma (PRP). 190 µl PRP with a platelet concentration of 2.5 × 10^5^/µl was stimulated with 10 µl ADP (0.2 mM) and light transmission was assessed as function over time using a light transmission aggregometer (möLAB).

### Assessment of bleeding time

Bleeding time was assessed upon 3 mm tail tip amputation under anesthesia and analgesia induced by i.p. 100 mg/kg ketamine (Zoetis) and 20 mg/kg xylazine (Bayer Vital). The remaining tail was immersed in saline at 37 °C and bleeding patterns were continuously monitored. Time was recorded until bleeding stopped.

### Generating cell suspensions for flow cytometry

Organ and tissue processing: After drawing venous blood by tail vein puncture, mice were sacrificed to harvest femur, tibiae and pelvis for BM and the heart for the infarcted myocardium. Venous blood was collected in 5 mM EDTA (Sigma-Aldrich) and lysed in 1 × red blood cell lysis buffer (BioLegend) prior to staining. Flushed bone marrow was passed through a 40 µm cell strainer to obtain a single cell suspension and collected in phosphate-buffered saline (PBS) containing 0.5% bovine serum albumin and 1% fetal bovine serum (FACS buffer). Infarcted myocardium was excised using a microscope, minced with scissors and digested in collagenase I (450 U/ml), collagenase XI (125 U/ml), DNase I (26 U/ml) and hyaluronidase (60 U/ml) (all Sigma-Aldrich). The mixture was incubated at 37 °C at 600 rpm for 1 h. The digestion reaction was stopped using 30 ml of FACS buffer.

### ADP / IL-1β / TNNI3 ELISA

Venous blood was acquired by tail vein puncture in potassium-EDTA microtubes (Sarstedt) and spun down for 8 min at 3.000 G to retrieve plasma. 50 µl plasma was used to measure levels of ADP and IL-1β, using ADP assay kit (Abcam) and Quantikine ELISA Mouse IL-1β (R&D) according to the manufacturers’ protocols. For bone marrow (BM), femoral bones were flushed in 5 mM EDTA (Sigma-Aldrich), spun down for 5 min at 4.000 G and supernatant was used for ADP ELISA (Abcam). For TNNI3-ELISA, blood was acquired by tail vein puncture in potassium-EDTA microtubes (Sarstedt) and spun down for 15 min at 1.000 G to retrieve plasma. 100 µl of 1:10 diluted plasma was used to measure protein levels of TNNI3, using Mouse TNNI3/Cardiac Troponin I kit (Life Span Biosciences) following the manufacturer’s protocol.

### CFU-assay with ADP stimulation

Colony forming unit (CFU) assays were performed according to the manufacturer’s protocol using a semi-solid cell culture medium (Methocult M3434, Stem Cell Technologies). Bones were flushed with PBS, supplemented with 0.5% bovine serum albumin and 2 mM EDTA. To deplete megakaryocytes and platelets, we used MACS depletion columns (LD columns, Miltenyi Biotec) after incubation with anti-CD41 (FITC, clone MWReg30, BioLegend) antibody, followed by an incubation with anti-FITC-coated microbeads (Miltenyi Biotec). 4.5 × 10^5^ BM cells were stimulated with 45 µl ADP (0.2 mM) in 3 ml medium to a final ADP concentration of 2.5–3 µM before they were plated onto six well plates in duplicates (1.5 × 10^5^ stimulated BM cells per well) for whole BM CFU-assay, whereas FACS-sorted LSK cells were used for LSK-specific CFU-assay. After 7 days of incubation, colonies were counted and analyzed using a low magnification inverted microscope.

### Quantitative real-time PCR

Using RNeasy Mini or Micro Kit (Qiagen), mRNA was extracted from flushed BM cells, platelets from PRP and infarcted myocardium according to manufacturers’ protocols. mRNA was then transcribed to cDNA using the High Capacity cDNA Reverse Transcription kit (Applied Biosystems). Using ARCTURUS pico pure RNA isolation kit (Applied Biosystems), mRNA was extracted from FACS-sorted cells and was further amplified and transcribed to cDNA with Ovation Pico SL WTA-System (NuGEN) according to the following protocol: first strand cDNA synthesis including primer annealing 65 °C for 2 min and first strand synthesis 4 °C for 2 min, 25 °C for 30 min, 42 °C for 15 min and 70 °C for 15 min, second strand cDNA synthesis 4 °C for 1 min, 25 °C for 10 min, 50 °C for 30 min and 80 °C for 20 min and single primer isothermal amplification 4 °C for 1 min, 47 °C for 75 min and 95 °C for 5 min.

Real-time PCR reactions used TaqMan Fast Advanced Master Mix (Applied Biosystems) and were run on a Thermal Cycler (BioRad). The following genes were analyzed with TaqMan Gene Expression Assays: GAPDH (Mm99999915_g1), GPIab (Mm00501677_g1), GPV (Mm00515021_s1), GPVI (Mm01332306_m1), ITGB 3 (Mm00443980_m1), P2RY12 (Mm01950543_s1), Cxcl12 (Mm00445553_m1), Kitl (Mm00442972_m1), Vcam1 (Mm01320970_m1), Angpt1 (Mm00456503_m1), TNFα (Mm00443258_m1), IL-1β (Mm00434228_1), MMP9 (Mm0044299_m1), TIMP1 (Mm01341361_m1) (all Applied Biosystems). Results were expressed as Ct values normalized to the housekeeping gene GAPDH (control was set as 1).

### Flow cytometry

The cell suspensions were resuspended in 300 µl FACS buffer (5 ml tube, Falcon) for BM and blood samples, 400 µl FACS buffer for MI samples and stained with fluorochrome-labelled antibodies as described below.

### Hematopoietic stem and progenitor staining

We first incubated cells with PE-conjugated anti-mouse antibodies directed against CD11b (clone M1/70), CD19 (clone 6D5), CD90.2 (clone 53-2.1), CD11c (clone N418), CD4 (clone GK1.5), CD8a (clone 53-6.7), CD127 (clone A7R34), CD49b (clone DX5), Ly-6G (clone 1A8), Ly-6C (clone HK1.4), TER-119 (clone TER-119) (all BioLegend). Then cells were stained with antibodies directed against c-kit (BioLegend, clone 2B8), sca-1 (BioLegend, clone D7), CD34 (BD Bioscience, clone RAM34), CD16/32 (BioLegend, clone 93), CD115 (eBioscience, clone AFS98). The term LSK refers to hematopoietic stem and progenitor cells based on their characteristic surface expression pattern (Lineage ^neg^, Sca-1^+^, c-Kit^+^) [42], specified as Lin (CD11b, CD19, CD90.2, CD11c, CD4, CD8a, CD127, CD49b, Ly-6G, Ly-6C, TER-119) ^low^, sca-1 ^high^, c-kit ^high^ and was used throughout the manuscript as it best describes the cell population investigated. Granulocyte–macrophage precursors (GMP) were defined as Lin (CD11b, CD19, CD90.2, CD11c, CD4, CD8a, CD127, CD49b, Ly-6G, Ly-6C, TER-119) ^low^, c-kit ^high^, sca-1 ^low^, (CD34/CD16/32) ^high^, CD115 ^int/low^. Monocyte-dendritic cell precursor (MDP) were defined as Lin (CD11b, CD19, CD90.2, CD11c, CD4, CD8a, CD127, CD49b, Ly-6G, Ly-6C, TER-119) ^low^, c-kit ^int/high^, sca-1 ^low^, (CD34/CD16/32) ^high^, CD115 ^high^ [25].

### Blood leukocyte staining

Cells were stained with CD45.2 (clone 104), CD19 (clone 6D5), CD3 (clone 17A2), CD11b (clone M1/70), CD115 (clone AFS98), Ly-6G (clone 1A8), and Ly-6C (clone HK1.4) (all BioLegend). Leukocytes were identified as CD45 ^high^. Myeloid cells were identified as CD45 ^high^ CD19 ^low^ CD3 ^low^ CD11b ^high^. Neutrophils were identified as CD45 ^high^ CD19 ^low^ CD3 ^low^ CD11b ^high^ CD115 ^low^ Ly-6G ^high^. Inflammatory monocytes were identified as CD45 ^high^ CD19 ^low^ CD3 ^low^ CD11b ^high^ Ly-6G ^low^ CD115 ^high^ Ly-6C ^high^. B-Lymphocytes were identified as CD45 ^high^ CD19 ^high^ CD3 ^low^.

### Leukocyte staining in the infarcted myocardium

We first incubated cells with PE-conjugated anti-mouse antibodies directed against CD19 (clone 6D5), CD90.2 (clone 53-2.1), CD4 (clone GK1.5), CD8a (clone 53-6.7), NK1.1 (clone PK136), TER-119 (clone TER-119), CD49b (clone DX5) (all BioLegend). Then cells were stained with antibodies directed against CD45.2 (clone 104), CD11b (clone M1/70), Ly-6G (clone 1A8), Ly-6C (clone HK1.4) and F4/80 (clone BM8) (all BioLegend). Leukocytes were identified as CD45 ^high^. Myeloid cells were identified as CD45 ^high^ (CD19, CD90.2, CD4, CD8a, NK1.1, TER-119, CD49b) ^low^ CD11b ^high^. Neutrophils were identified as CD45 ^high^ (CD19, CD90.2, CD4, CD8a, NK1.1, TER-119, CD49b) ^low^ CD11b ^high^ Ly-6G ^high^. Inflammatory monocytes were identified as CD45 ^high^ (CD19, CD90.2, CD4, CD8a, NK1.1, TER-119, CD49b) ^low^ CD11b ^high^ Ly-6G ^low^ Ly-6C ^high^ F4/80 ^low^. Macrophages were identified as CD45 ^high^ (CD19, CD90.2, CD4, CD8a, NK1.1, TER-119, CD49b) ^low^ CD11b ^high^ Ly-6G ^low^ Ly-6C ^low^ F4/80 ^high^.

Data were acquired using FACS Canto™ II, LSRFortessa™ and FACS Diva software (BD Pharmingen). Experimental data were analyzed using FlowJo software.

### Intracellular staining

Cell cycle analysis was performed as previously described [25] using intranuclear Ki67 (eBioscience, clone SolA15) and DAPI (4,6-diamidino-2-phenyl-indole, FxCycle Violet Stain, Life Technologies) staining, or APC/FITC BrdU flow kits (BD Pharmingen). 1 mg BrdU was injected intraperitoneally 24 h prior to organ harvest. Ki67 / DAPI staining and BrdU staining were performed after staining of cell surface markers according to the manufacturer’s protocol.

For Akt intracellular staining, we used anti-mouse antibodies directed against phospho-Akt (S473) (monoclonal mouse IgG_1_ clone # 545,007) and pan-Akt (monoclonal mouse IgG_2B_ clone # 281,046).

### Cell sorting

For cell sorting of LSK cells and myeloid progenitors (GMP + MDP), BM cells were collected from individual mice by flushing bones from both femurs, tibias and the pelvis and stained as described above. For sorting myeloids and lymphocytes, blood leukocytes staining was performed as described above. Adding anti-CD61 antibody (clone 2C9.G2 (HMβ3-1)) to the lineage allowed platelet and megakaryocyte exclusion. FACS-sorting was performed by FACS Aria III and Fusion cell sorter (BD Pharmingen).

### Histology

For immunohistochemistry, hearts were harvested and embedded in O.C.T compound (Sakura Finetek). Embedded tissues were snap-frozen in dry ice. Sections of 5 µm thickness were then stained using antibodies directed against CD11b (clone M1/70) or CD31 (clone MEC 13.3) (all BioRad). Staining was followed with a biotinylated secondary antibody. We used the VECTASTAIN Elite ABC HRP kit and ImmPACT AMEC Red Peroxidase (HRP) substrate (Vector Laboratories, Inc.) for color development. For Masson’s trichrome staining, we used Weigert’s iron hematoxylin solution and Accustain Trichrome Stain Kit (both Sigma-Aldrich) according to the manufacturer’s protocol.

### Echocardiography

Echocardiography was performed as previously described [40] to assess left ventricular ejection fraction, end-systolic and end-diastolic volume and stroke volume.

### Statistics

Statistical analyses were carried out using GraphPad Prism software version 8 (GraphPad Software, Inc.). Results are displayed as mean ± standard error of mean (S. E. M.). First, values were tested for Gaussian distribution (D’Agostino-Pearson omnibus normality test). For two-group comparisons, unpaired t-test was applied to parametric data, Mann–Whitney test to non-parametric data. For comparing more than two groups an ordinary one-way ANOVA test followed by a Sidak’s test for multiple comparisons was applied to parametric data; the Kruskal–Wallis test was applied to non-parametric data. *P* values of < 0.05 indicated statistical significance.

## Results

### Increased ADP levels in the BM after MI stimulate hematopoiesis via P2Y_12_ receptor dependent activation of the Akt signaling pathway in LSK cells

**Fig. 1:**
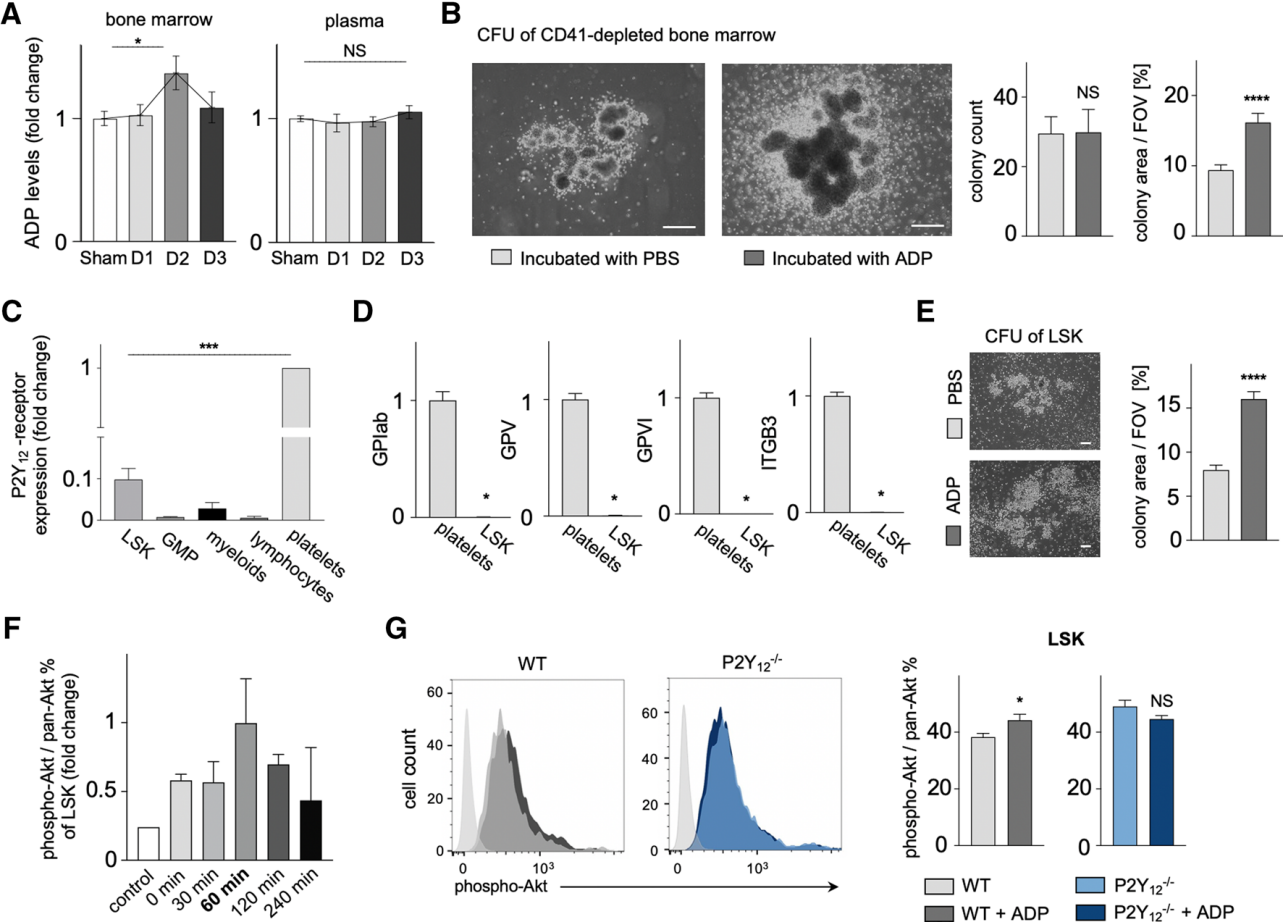
**A** Timeline of ADP levels in the bone marrow and plasma, assessed by ELISA on day 1, 2 and 3 after MI in comparison to sham-operated C57BL/6 mice (*n* = 12–40 per group; Kruskal–Wallis test for BM, one-way ANOVA for plasma). **B** CFU-assay performed with flushed bone marrow cells from C57BL/6 WT mice after CD41 depletion. Bar graphs illustrate macroscopic colony count (*n* = 6–8 per group; Mann–Whitney test) and microscopic colony area per field of view (FOV) in % (*n* = 60–77 per group; Mann–Whitney test). Scale bar indicates 500 µm. **C** Relative expression of the ADP receptor P2Y_12_ in different cell types, evaluated by qPCR from bone marrow cell populations sorted by FACS under CD41 exclusion (*n* = *6* per group; Kruskal–Wallis test). **D** Platelet-specific markers GPIab, GPV, GPVI and ITGB3 from FACS-sorted LSK cells, evaluated by qPCR and shown as fold change (*n* = 4 per group; Mann–Whitney test). **E** CFU-assay performed with FACS-sorted LSK cells after exclusion of CD41^+^ cells. The bar graph illustrates microscopic colony area per FOV in % (*n* = 30 per group; student's *t* test). Scale bar indicates 500 µm. **F** Timeline of Akt signaling pathway activation in LSK cells in vitro, assessed by flow cytometry 0 min, 30 min, 60 min, 120 min and 240 min after ADP (1 µM) stimulation compared to control (no ADP), shown as fold change of phospho-Akt to pan-Akt ratio (*n* = 2–3 per group). **G** Histograms illustrates Akt signaling pathway activation in LSK cells in vitro 60 min after ADP (1 µM) stimulation in C57BL/6 wildtype (WT) (left) and P2Y_12_^−/−^ mice (right) in comparison to unstained control (light grey). Bar graphs show phospho-Akt to pan-Akt ratio in LSK from WT and P2Y_12_^−/−^ mice (*n* = 5 per group; Mann–Whitney test). Mean ± S.E.M., *p < 0.05, **p < 0.01, ***p < 0.001, ****p < 0.0001

Screening for danger signals after acute ischemic myocardial injury induced by permanent coronary ligation, we found elevated ADP levels in the hematopoietic BM on day 2 after MI, whereas no change was observed in plasma when compared to sham surgery (Fig. 1A). As the recorded peak of ADP in the BM coincided with the known time point for maximum post-ischemic activation of hematopoietic stem and progenitor cells, we investigated if ADP could serve as a direct messenger to the BM for emergency hematopoiesis. We performed in vitro colony forming unit (CFU) assays incubating BM cells of wildtype (WT) mice with ADP (2.5–3 µM) or PBS. Prior to stimulation, CD41^+^ cells were depleted to avoid ADP-dependent release of secondary messengers by platelets. After 7 days, we found similar absolute numbers of CFUs on each plate, but most notably, significantly larger colonies on plates with ADP-stimulated BM as compared to PBS-control (Fig. 1B).

In search of a plausible explanation for the responsiveness of the BM to the elevated post-ischemic ADP levels, we isolated an array of hematopoietic cell populations by flow cytometric sorting, i.e. LSK cells, downstream hematopoietic progenitors (granulocyte macrophage progenitors, GMP) and differentiated immune cells (platelets, myeloid cells, lymphocytes) from the BM and peripheral blood to assess for the expression of the ADP receptor P2Y_12_. Beyond the well appreciated P2Y_12_ expression on platelets, we found relevant mRNA expression of P2Y_12_ on LSK cells (Fig. 1C), while mRNA of other platelet-specific markers, i.e. GPIab, GPV, GPVI and ITGB3, was not detectable (Fig. 1D). Performing CFU assays only with isolated LSK cells, we confirmed larger colonies on ADP (2.5–3 µM)-stimulated plates compared to the PBS control (Fig. 1E). Since most current RNA sequencing databases of the hematopoietic BM do not describe P2Y_12_ receptor expression on LSK cells, we matched our findings with an existing RNA-sequencing repository of enriched hematopoietic stem and progenitor cells [33]. Here, we were able to confirm detectable P2Y_12_ receptor expression on LSK cells in comparison to Lineage^−^ sca-1^−^ c-kit^+^ progenitor cells (Supp. Fig. S1).

For further characterization and functional evidence of the P2Y_12_ receptor on LSK cells, we analyzed the intracellular Akt signaling pathway, which is known to be activated downstream of P2Y_12_ and to be involved in cell cycle progression. We found Akt phosphorylation, evaluated as the phospho-Akt to pan-Akt ratio, to increase in response to ADP and peak 60 min upon ADP (1 µM) stimulation in WT LSK cells (Fig. 1F), while no change was observed in LSK cells of P2Y_12_ deficient mice (Fig. 1G).

### P2Y_12_ receptor-deficiency blocks ADP-dependent cell expansion and reduces cell cycle progression of LSK cells in the BM after MI

**Fig. 2:**
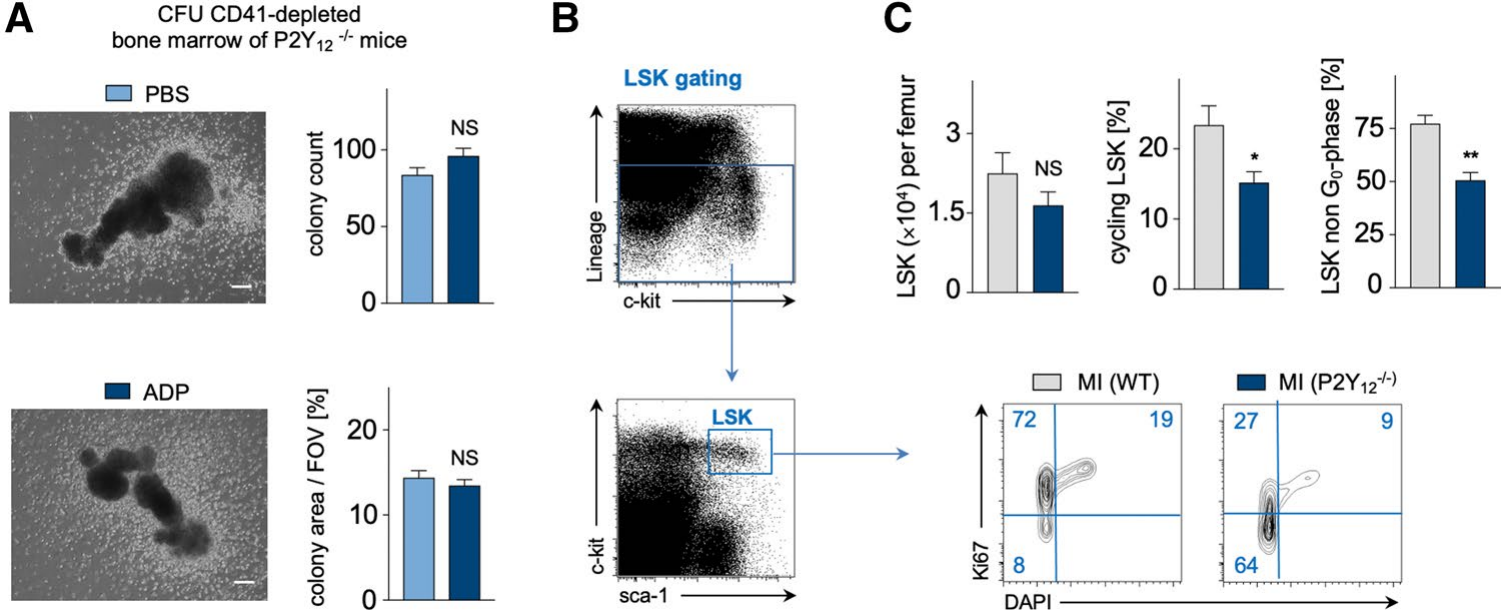
**A** CFU-assay performed with flushed bone marrow cells from C57BL/6 P2Y_12_^−/−^ mice after CD41 depletion. Bar graphs illustrate macroscopic colony count (*n* = 8 per group; student’s *t* test) and microscopic colony area per field of view (FOV) in % (*n* = 78–80 per group; Mann–Whitney test). Scale bar indicates 500 µm. **B** Flow cytometric gating for LSK cells. **C** Cell cycle analysis performed with Ki67 / DAPI assay in C57BL/6 WT and P2Y_12_^−/−^ mice on day 2 after MI. Bar graphs show absolute numbers of LSK cells per femur, LSK cycling and non G_0_-phase rates in % on day 2 after MI (*n* = 7–12 per group; Mann–Whitney test). Mean ± S.E.M., *p < 0.05, **p < 0.01

Assuming that ADP stimulates the expansion of hematopoietic BM after MI via P2Y_12_ signaling, we hypothesized BM of P2Y_12_-deficient (−/−) mice to remain unresponsive to ADP. In fact, in vitro CFU assays with BM of P2Y_12_^−/−^ mice showed no significant difference in colony size after stimulation with ADP (2.5–3 µM) compared to PBS-control (Fig. 2A). Finally, we compared the BM response of WT and P2Y_12_^−/−^ mice in vivo on day 2 after MI. LSK populations were identified as shown in Fig. 2B. With similar numbers of total LSK cells in the BM, LSK cells of P2Y_12_^−/−^ mice were significantly less activated and presented lower cell cycle activity and a higher fraction of LSK remaining in G_0_-phase after MI compared to WT mice after MI (Fig. 2C).

### Treatment with the P2Y_12_ inhibitor prasugrel reduces cell cycle entry of LSK cells and numbers of downstream hematopoietic progenitors in the BM after MI

**Fig. 3:**
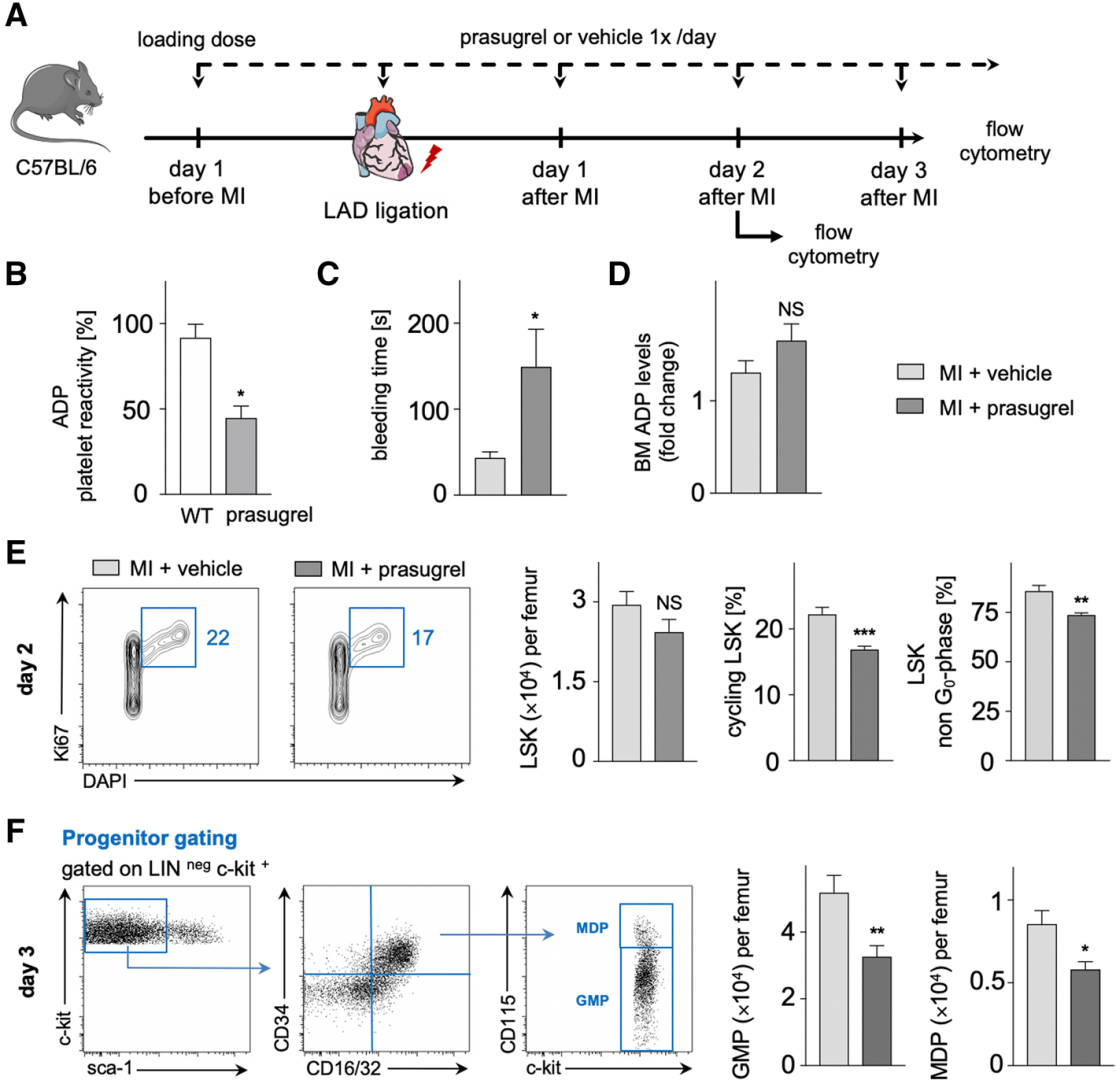
**A** Schematic illustration of the experimental setup. After establishing P2Y_12_ receptor inhibition by a loading dose of prasugrel, LAD was ligated for MI and analysis was performed on day 2 and 3 after MI as shown. **B** Platelet reactivity after 2 days of oral prasugrel treatment in comparison to wildtype, measured by light transmission aggregometry (*n* = 4 per group; Mann–Whitney test). **C** Bleeding time under prasugrel treatment versus vehicle control (*n* = 6 per group; Mann–Whitney test). **D** ADP levels in the BM on day 2 after MI in prasugrel-treated C57BL/6 mice compared to vehicle control, assessed by ELISA (*n* = 10 per group; unpaired *t* test). **E** Cell cycle analysis of LSK cells performed with Ki67/DAPI assay in prasugrel-treated C57BL/6 mice in comparison to vehicle control. Bar graphs show LSK cell numbers per femur, LSK cycling rates and portion of LSK in G_2_/S/M phase (non G_0_) in % on day 2 after MI (*n* = 7–11 per group; student’s *t* test for LSK cell numbers per femur and LSK cycling rates, Mann–Whitney test for LSK non G_0_-phase). **F** Flow cytometric gating for downstream hematopoietic progenitor populations GMP and MDP. Bar graphs show GMP and MDP numbers per femur in prasugrel-treated C57BL/6 mice in comparison to vehicle control on day 3 after MI (*n* = 16–19 per group; student’s *t* test). Mean ± S.E.M., *p < 0.05, **p < 0.01, ***p < 0.001

Genetic mouse models are susceptible to unknown intrinsic phenotypes due to gene editing. Therefore, we investigated emergency hematopoiesis after MI with therapeutic drug targeting of the P2Y_12_ receptor using prasugrel as a potent, specific and irreversible P2Y_12_ antagonist that is commonly used in patients with ST-segment elevation MI. The treatment protocol was performed as illustrated in Fig. 3A. Efficacy of the treatment regimen was confirmed by platelet reactivity tests to ADP-stimulation (Fig. 3B) and assessment of tail bleeding time (Fig. 3C). Notably, prasugrel treatment did not influence ADP levels in the BM (Fig. 3D). As observed in P2Y_12_^−/−^ mice, we found similar numbers of total LSK cells, but significantly reduced entry of LSK cells into the cell cycle in mice with prasugrel treatment in comparison to vehicle on day 2 after MI (Fig. 3E). These findings were validated in a BrdU incorporation assay showing significantly reduced BrdU uptake in LSK cells of prasugrel-treated mice after MI (Suppl. Fig. S2). Furthermore, we observed reduced numbers of downstream myeloid precursors, i.e. GMP and MDP, in the BM of mice treated with prasugrel as compared to vehicle on day 3 after MI (Fig. 3F).

### P2Y_12_ receptor inhibition with prasugrel does not influence levels of pro-inflammatory IL-1β, expression of BM niche factors nor mobilization of hematopoietic progenitors after MI

To put the described ADP-dependent, P2Y_12_-mediated signaling pathway into the context of the multilayered inflammatory response to MI, we examined established danger signals for the hematopoietic BM after MI. Prasugrel treatment did not affect the concentration of IL-1β in the blood on day 1 after MI (Supp. Fig. 3A), the expression of regulatory BM retention factors, known to influence homeostasis and activation of the hematopoietic BM after MI (Supp. Figure 3B) nor release of hematopoietic stem and progenitor cells from the BM to the peripheral blood (Supp. Fig. 3C).

### Cyclooxygenase (COX)-mediated platelet inhibition by acetylsalicylic acid (ASA) does not influence LSK cell cycle activity nor cell numbers in the BM after MI

Since the observed effects of P2Y_12_-deficiency or prasugrel treatment on emergency hematopoiesis could also be related to platelet inhibition, we analyzed the hematopoietic response to ischemic myocardial injury by inhibiting another common pathway for platelet activation, the cyclooxygenase-thromboxane pathway, using acetylsalicylic acid (ASA) (Supp. Fig. 4A). Platelet inhibition was confirmed by assessment of functional tail bleeding time after ASA treatment (Supp. Fig. 4B). On day 2 after MI, treatment with ASA did not change the rate of cycling LSK in the BM nor the fraction of cells in the G_0_-phase of the cell cycle. Importantly, no difference in downstream myeloid progenitors was observed (Supp. Fig. 4C).

### P2Y_12_ receptor inhibition reduces numbers of inflammatory leukocytes in the blood and the infarcted myocardium after MI

**Fig. 4:**
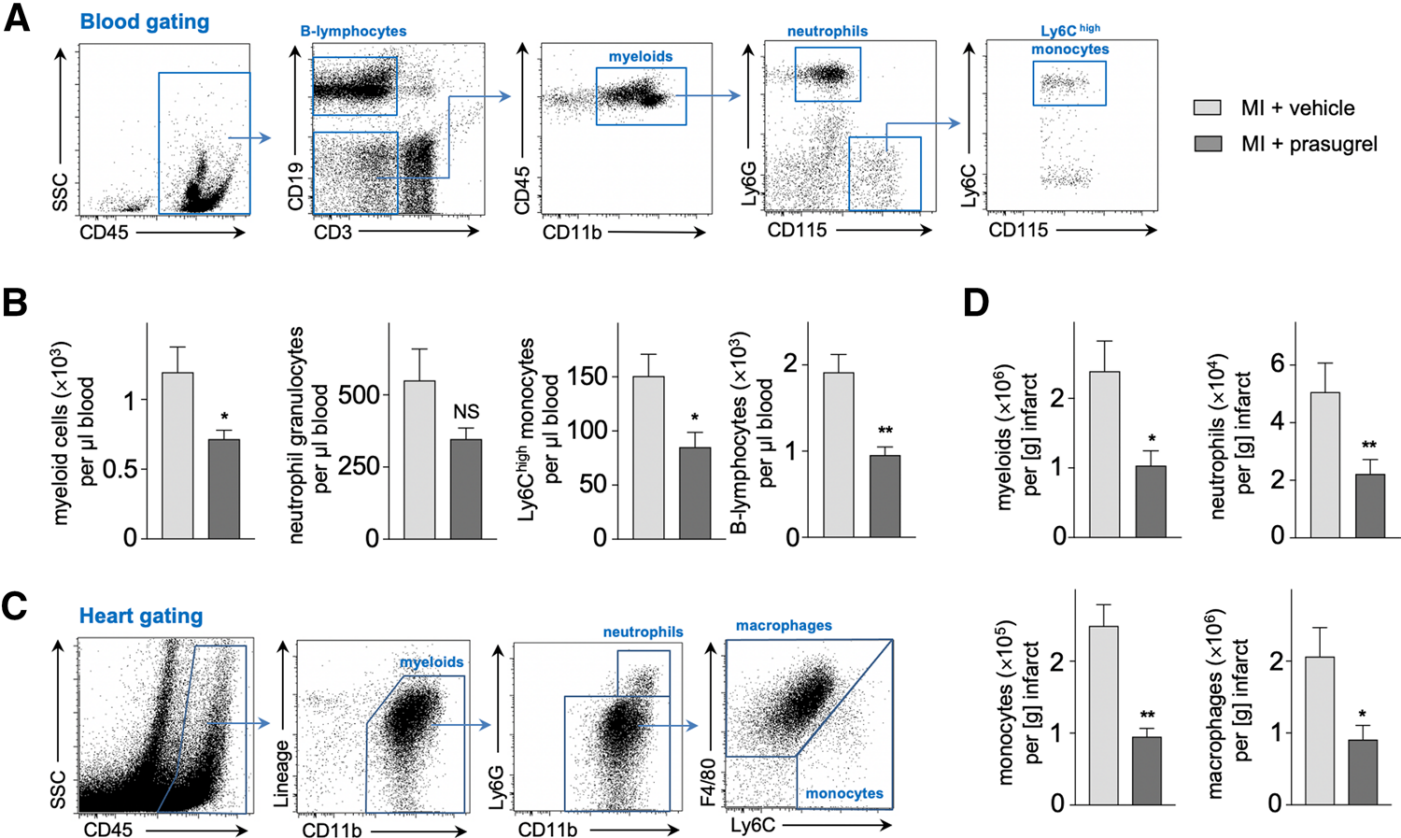
**A** Flow cytometric gating for B-lymphocytes, myeloids, neutrophils and Ly6C ^high^ monocytes in the blood. **B** Effects of P2Y_12_ receptor blocker prasugrel on blood leukocytes on day 3 after MI (*n* = 8–9 per group; unpaired *t* test). Bar graphs display cell counts per µl blood. **C** Flow cytometric gating for myeloids, neutrophils, monocytes and macrophages in the infarcted myocardium. **D** Leukocytes and subsets in the infarcted myocardium on day 7 after MI in prasugrel-treated C57BL/6 mice compared to vehicle control, assessed by flow cytometry (*n* = 6–7 per group; Mann–Whitney test). Bar graphs show cell counts per g of infarcted myocardium. Mean ± S.E.M., *p < 0.05, **p < 0.01

As emergency hematopoiesis in upstream hematopoietic progenitors is ultimately linked to leukocyte production, we evaluated the composition of the inflammatory response to injury under P2Y_12_ receptor inhibition with prasugrel on day 3 after MI. Blood leukocyte subpopulations were identified as shown in Fig. 4A. Treatment with prasugrel significantly reduced numbers of innate immune cells, namely myeloids cells, neutrophils and inflammatory Ly6C^high^ monocytes and also B-lymphocytes in the blood (Fig. 4B). Furthermore, P2Y_12_ inhibition with prasugrel also affected recruitment of inflammatory immune cells to the infarct, which is a known prognostic parameter for wound healing and cardiac remodeling. Leukocytes in the infarct and border zone were identified as shown in Fig. 4C. On day 7 after MI, prasugrel-treated mice showed significantly reduced infiltration of myeloid cells, including neutrophils, monocytes and macrophages in comparison to vehicle (Fig. 4D).

### The P2Y_12_ receptor-mediated effects on LSK cells after MI are independent from platelets

**Fig. 5:**
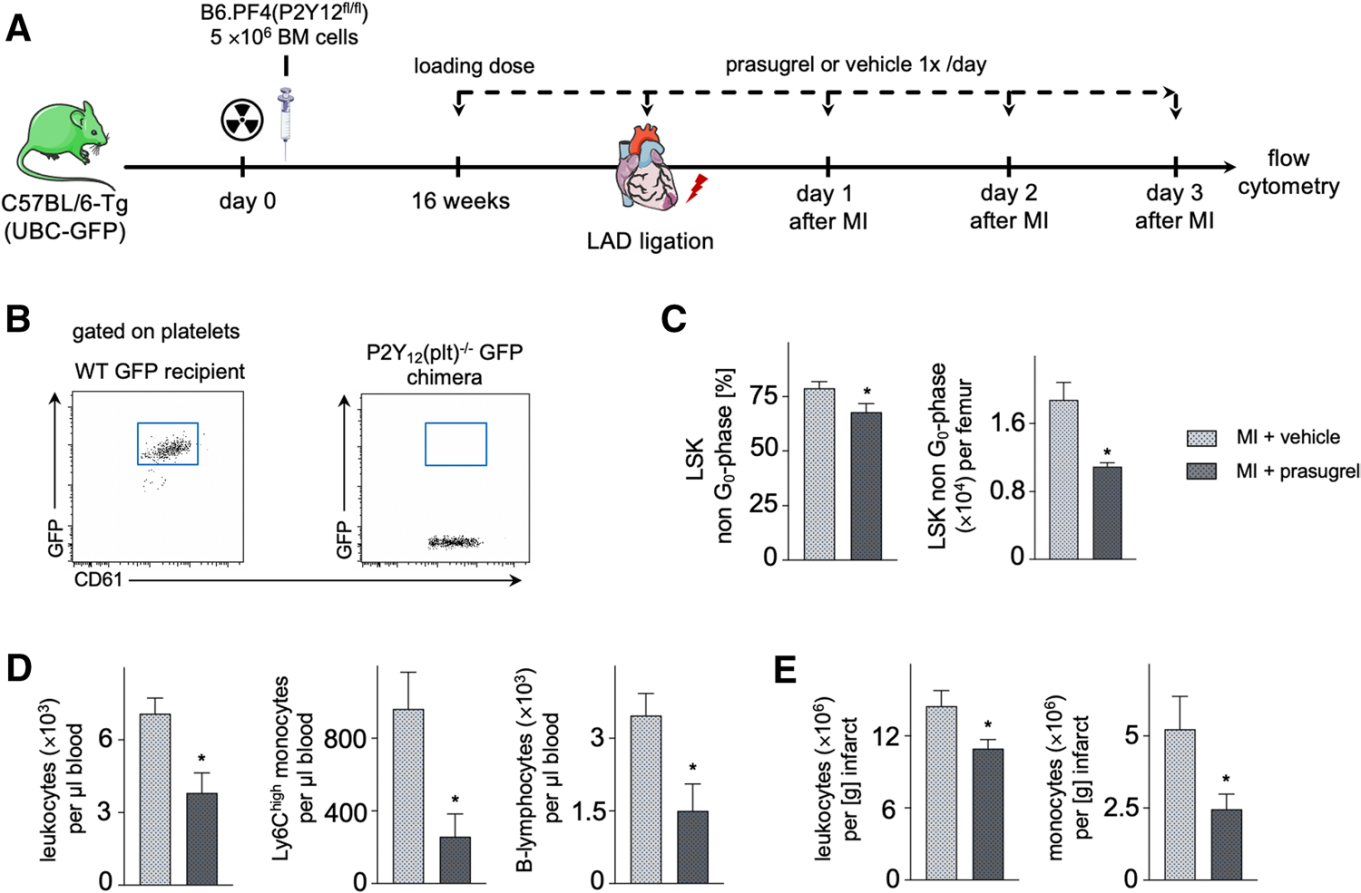
**A** Schematic illustration of the experimental setup. C57/BL/6-Tg (UBC-GFP) mice were lethally irradiated and reconstituted with PF4(P2Y_12_^fl/fl^) BM cells to create P2Y_12_(plt)^−/−^ GFP chimeras. After 16 weeks, LAD was ligated for MI and analysis was performed on day 3 after MI as shown. **B** Residual GFP^+^ platelets in P2Y_12_(plt)^−/−^ GFP chimeras compared to GFP WT recipients. **C** Proliferation analysis of LSK cells in prasugrel-treated P2Y_12_(plt)^−/−^ GFP chimeras in comparison to vehicle control. Bar graphs show the portion of LSK cells in G_1_/G_2_/S/M phase (non G_0_) in % and numbers of LSK cells in non G_0_ phase per femur on day 3 after MI (*n* = 5–6 per group; Mann–Whitney test). **D** Effects of P2Y_12_ receptor blocker prasugrel on blood leukocytes on day 3 after MI in P2Y_12_(plt)^−/−^ GFP chimeras (*n* = 6 per group; Mann–Whitney test). Bar graphs display cell count per µl blood. **E** Leukocytes and Ly6C^high^ monocytes in the infarcted myocardium on day 3 after MI in prasugrel-treated P2Y_12_(plt)^−/−^ GFP chimeras in comparison to vehicle control, assessed by flow cytometry (*n* = *6* per group; Mann–Whitney test). Bar graphs show cell counts per g of infarcted myocardium. Mean ± S.E.M., *p < 0.05

Platelets harbor immunoregulatory functions that may as well be targeted by ubiquitous P2Y_12_ knockout or prasugrel treatment. We created platelet-specific P2Y_12_-deficient GFP chimera (P2Y_12_(plt)^−/−^ GFP) to investigate the contribution of platelet-P2Y_12_ to the described effects on hematopoietic BM expansion and post-MI inflammation. The transplantation and treatment protocols were performed as illustrated in Fig. 5A. P2Y_12_(plt)^−/−^ GFP chimera showed no residual presence of GFP^+^ P2Y_12_-competent platelets (Fig. 5B).

On day 3 after MI, treatment of with prasugrel significantly reduced LSK cell cycling in the bone marrow (Fig. 5C), which translated to reduced numbers of leukocytes in circulation (Fig. 5D) and the infarcted myocardium (Fig. 5E) as compared to P2Y_12_(plt)^−/−^ GFP chimeras treated with vehicle control.

### Inhibition of the P2Y_12_ receptor ameliorates chronic adverse cardiac remodeling and preserves cardiac function 3 weeks after MI

**Fig. 6:**
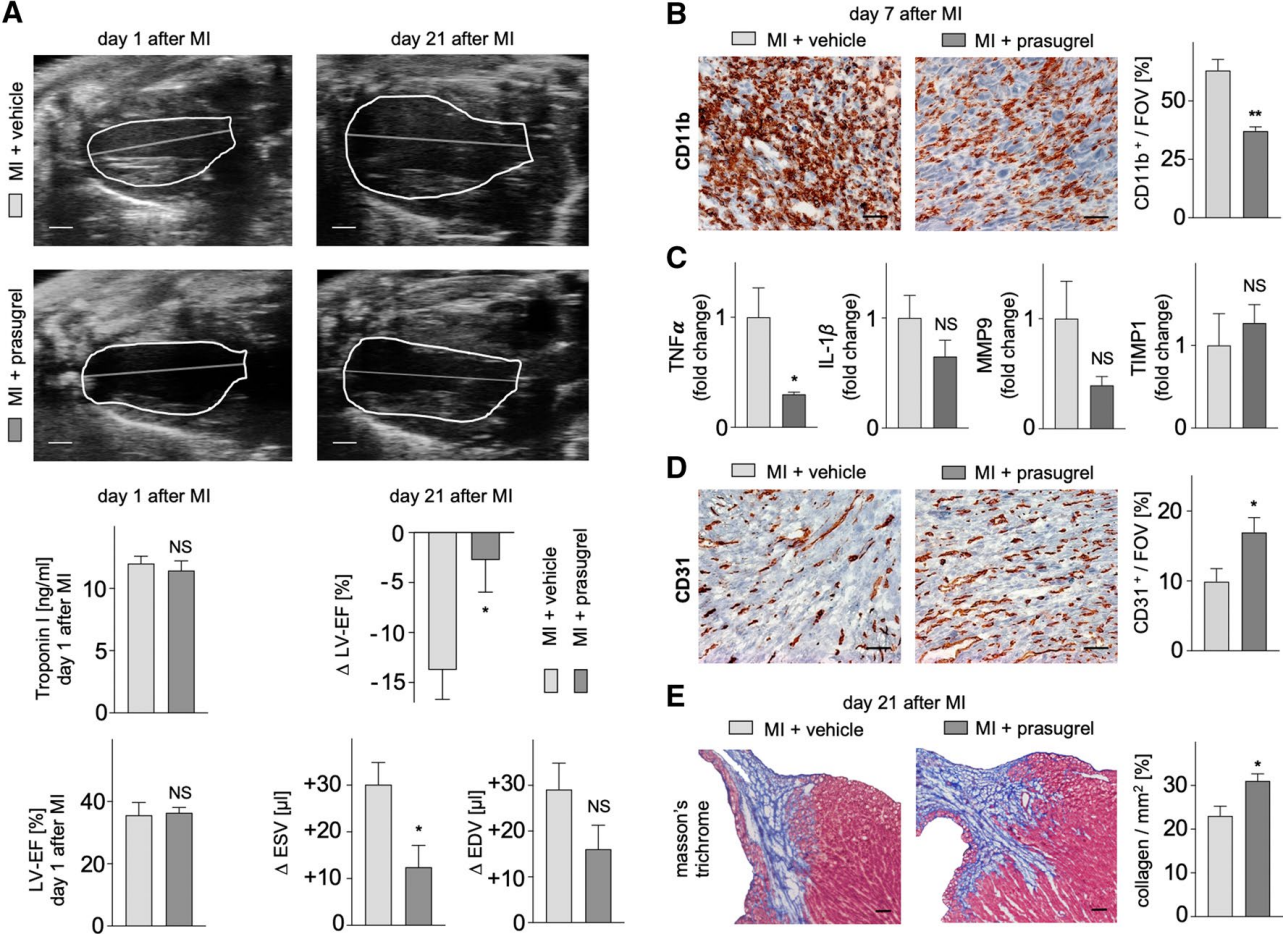
**A** Evaluation of cardiac function and volumes by echocardiography day 1 and day 21 after MI in C57BL/6 mice under prasugrel treatment compared to vehicle control until day 7 after MI. Depicted are end-systolic parasternal long axis views in B-mode. Scale bar indicates 1 mm. Bar graphs show troponin I levels in plasma on day 1 after MI, evaluated by ELISA (*n* = 7–8 per group, Mann–Whitney test), left ventricular ejection fraction (LV-EF in %) on day 1 after MI (*n* = 7–8 per group, Mann–Whitney test) and delta changes in left ventricular ejection fraction, end-systolic and end-diastolic volumes (∆ LV-EF in %, ∆ ESV and EDV in µl) between day 1 and day 21 after MI (*n* = 7−8 per group, Mann–Whitney test) in prasugrel-treated C57BL/6 mice compared to vehicle control. **B** Immunohistochemistry for CD11b of the infarcted myocardium (border zone) on day 7 after MI. Scale bar indicates 50 µm. Bar graphs show percentages of the positive area for CD11b in the infarcted area per field of view in % (*n* = 6–8 per group; Mann–Whitney test). **C** TNF α, IL-1β*,* MMP9 and TIMP in the infarcted myocardium (border zone) on day 7 after MI in C57BL/6 mice under prasugrel treatment compared to vehicle control, evaluated by qPCR (*n* = 4–16 per group; unpaired *t* test for TNF α, IL-1β*,* MMP9, Mann–Whitney test for TIMP). **D** Immunohistochemistry for CD31 of the infarcted myocardium (border zone) on day 7 after MI. Scale bar indicates 50 µm. Bar graphs show percentages of the positive area for CD31 in the infarcted area per field of view in % (*n* = 5–8 per group; Mann–Whitney test). **E** Masson’s Trichrome staining of the infarcted myocardium (border zone) on day 21 after MI. Scale bar indicates 100 µm. Bar graphs show percentages of collagen in the infarcted area per mm^2^ (*n* = 7–8 per group; Mann–Whitney test). Mean ± S.E.M., *p < 0.05, **p < 0.01

Cardiac function and remodeling were recorded echocardiographically on day 1 and day 21 following MI in mice treated with prasugrel or vehicle control. Comparable size of the induced myocardial injury was validated by similar troponin I levels and equally reduced cardiac function in both groups on day 1 after MI (Fig. 6A). On day 21 after MI, animals treated with prasugrel showed superior preserved cardiac function and limited left ventricular dilatation, recorded as reduction of end-systolic left ventricular volume increment, compared to the vehicle group (Fig. 6A). These findings were supported by immunohistochemistry, showing reduced staining for myeloid CD11b^+^ cells (Fig. 6B).

Cardiac remodeling describes a delicately balanced process of post-ischemic inflammation and reparative wound healing. We evaluated mRNA expression of selected key players of cardiac remodeling in the border zone of the infarct on day 7 after MI which were reduced after prasugrel treatment (Fig. 6C). Interestingly, neovascularization by means of staining for CD31^+^ cells (Fig. 6D) and collagen content in Masson’s trichrome staining (Fig. 6E) in the border zone of the infarct were detected to be higher under P2Y_12_ receptor inhibition with prasugrel compared to vehicle. 

## Discussion

**Fig. 7:**
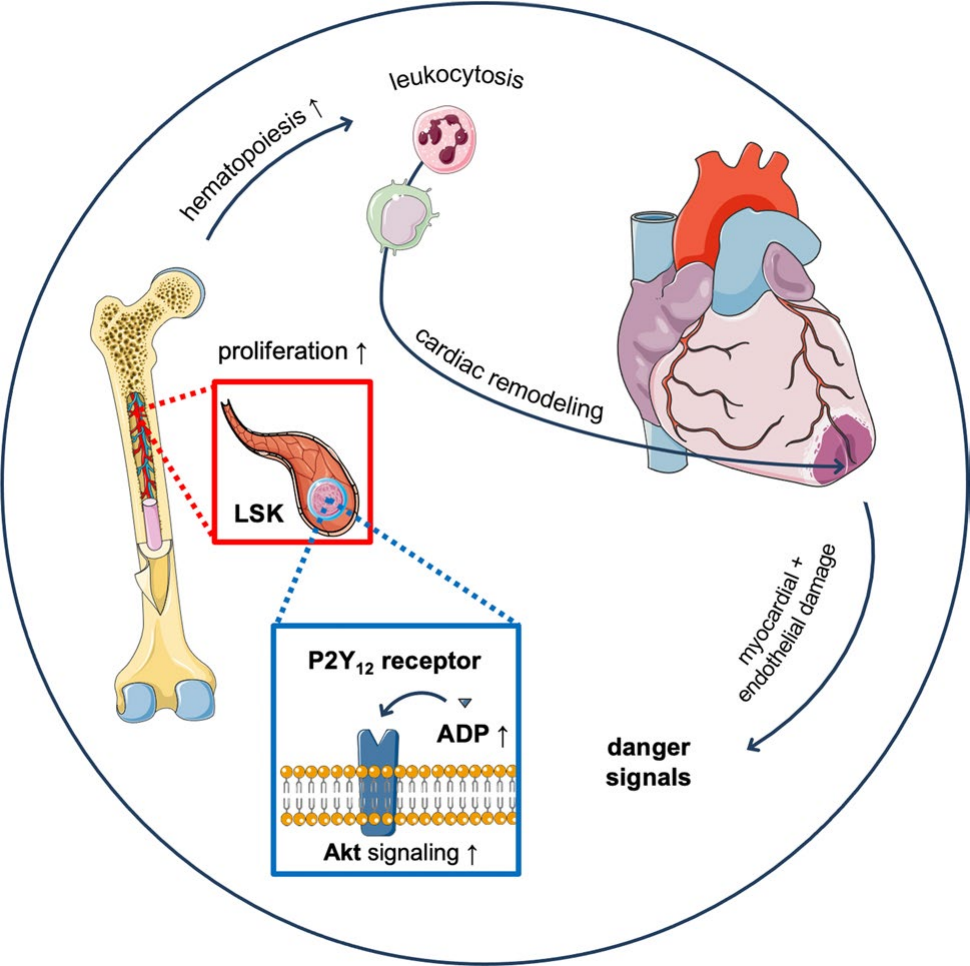
Summary

After MI, blood leukocyte counts correlate with in-hospital mortality and recurrent adverse cardiovascular events [21, 23]. Modulating the inflammatory response to ischemic myocardial injury has therefore been a promising approach to improve patients’ outcome after MI and has recently been in focus of several clinical trials on secondary cardiovascular prevention [45, 54, 55, 67]. In this study, we describe that the ADP-dependent, P2Y_12_ receptor-mediated signaling pathway is a key driving factor for emergency hematopoiesis after MI. We identified ADP as a danger signal for the hematopoietic BM since ADP levels increased after MI and promoted phosphorylation of Akt and cell cycle progression of hematopoietic stem and progenitor cells (LSK) in vitro. We detected P2Y_12_ receptor expression on LSK cells which implicates that ADP acts directly on LSK cells via P2Y_12_ signaling, not mediated by P2Y_12_ on platelets, which was confirmed in mice with platelet-specific P2Y_12_-deficiency. Ubiquitous P2Y_12_ knockout or treatment with the P2Y_12_ receptor antagonist prasugrel modulated emergency hematopoiesis, subsequently reducing the excessive inflammatory response to MI, which translated into reduced expansion of downstream lineages and limited the numbers of leukocytes in circulation and in the infarct. Ultimately, this preserved cardiac function and prevented adverse cardiac remodeling after MI (Fig. 7).

Acute ischemic myocardial injury triggers a profound release of danger-associated molecular patterns (DAMPs) and cytokines from activated platelets, injured cardiomyocytes and endothelial cells [51]. These danger signals then initiate a pro-inflammatory cascade that mediates local inflammation, steers leukocyte recruitment to the infarct [15, 20] and fosters emergency hematopoiesis in the bone marrow to meet the excessive demand for leukocytes [3, 6, 10, 12, 25, 65]. Extracellular nucleotides such as ATP, ADP and UDP are among the prominent danger signals released upon vascular injury [7]. Whereas ATP is most abundant upon hypoxia or tissue acidosis and can activate several P2 receptors [51, 74], ADP is primarily known to be secreted from platelets’ dense granula upon activation and to act as a ligand to the purinergic P2Y_12_ receptor. Purinergic P2 receptors are closely linked to the inflammatory cascade [24, 61]. Intriguingly, hematopoietic stem and progenitor cells have been reported to express P2X and P2Y receptors in mice and humans [13, 17, 35, 56, 73]. While P2X_7_ has previously been linked to mobilization and homing of hematopoietic stem cells [1], the role of the P2Y receptors on hematopoietic progenitors, especially the P2Y_12_ receptor, has not yet been characterized.

Most DAMPs are released instantly in the course of injury. Yet, we found ADP levels in the BM to peak specifically on day 2 after MI. Interestingly, this increase in ADP concentrations coincided with the onset of hematopoietic stem cell activation following ischemic myocardial injury as previously reported [10, 11, 57], suggesting that ADP possibly serves as a messenger to the BM in favor of emergency hematopoiesis. Indeed, stimulating hematopoietic BM cells with ADP in cell culture increased BM cell proliferation. Importantly, platelets were excluded during the cell selection process for this experiment as ADP-dependent platelet activation in culture could have confounded data via cytokine release.

Looking for possible sources of ADP in the BM, we primarily considered activated platelets as well-acknowledged origin of extracellular ADP in hemostasis. Platelet inhibition, however, did not significantly decrease ADP concentrations in the BM or blood. As an alternative source, conversion of ATP to ADP and AMP, which occurs within hours after release, could be discussed as a delayed source of ADP as a secondary messenger [64]. However, detecting ATP conversion to ADP after ischemia has been impeded due to the short half-life of ADP and ample presence of ectonucleotidases in vivo [53, 77], leaving the designated origin of ADP in the hematopoietic BM after MI still to be elucidated.

To identify possible recipients to the elevated ADP levels, we screened various hematopoietic cell populations for the expression of the most commonly known ADP receptor P2Y_12_. Beyond the well appreciated P2Y_12_ expression on platelets [28], several other cell types including microglia in the brain, osteoclasts, vascular smooth muscle cells, leukocytes and hematopoietic progenitor cells have been reported to express P2Y_12_ [8, 18, 63, 73]. However, the functional relevance of P2Y_12_ expression beyond platelet aggregation is largely unknown [14]. Remarkably, after careful exclusion of platelet-complexes in the cell isolation process, we detected P2Y_12_ receptor expression, specifically on hematopoietic stem and progenitor cells (LSK) in the murine BM. This finding could be validated interrogating an external RNA sequencing dataset of isolated LSK cells [33]. In line with previous studies, we were also able to detect P2Y_12_ expression on differentiated leukocyte subsets at lower expression levels as compared to LSK cells. Therefore, direct P2Y_12_ dependent effects on leukocyte subsets have to be acknowledged and remain to be investigated.

The phosphoinositide 3-kinase (PI3K)/Akt intracellular signaling pathway promotes cell survival, proliferation and growth by phosphorylation and inhibition of key transcription factors and has been reported to forward Akt phosphorylation downstream of the P2Y_12_ receptor in activated platelets and vascular smooth muscle cells [32, 48, 59]. Stimulating LSK cells with ADP, we detected increased Akt phosphorylation, which was absent in P2Y_12_-deficient LSK cells. Furthermore, performing LSK-specific CFU assays confirmed LSK as a protagonist cell population. The P2Y_12_ receptor was validated to primarily moderate the expansion of hematopoietic BM cells in response to ADP, as P2Y_12_-deficiency depleted the pro-proliferative effect of ADP. This minimized possible pleiotropic effects due to dephosphorylation of ADP to AMP and adenosine to be responsible for the effect on hematopoietic BM proliferation. Taken together, our experiments implicate a close relation of ADP-dependent P2Y_12_ signaling with Akt phosphorylation and cell cycle progression in LSK cells and appeared to be well in line with previous reports on Akt-dependent proliferation of LSK cells with primarily myeloid lineage differentiation [31].

We performed permanent coronary ligation to induce acute ischemic myocardial injury in a murine model to analyze the inflammatory response to MI in the setting of selective P2Y_12_ targeting, achieved by P2Y_12_-deficiency or pharmacological P2Y_12_ receptor inhibition. Permanent coronary ligation was preferred over an ischemia / reperfusion model to reduce the effect of local, platelet-mediated reperfusion injury after MI. For P2Y_12_ antagonist treatment, we chose the second generation thienopyridine prasugrel, which was reported to be more potent than clopidogrel in terms of more rapid and consistent P2Y_12_ inhibition [75]. Despite its promising favorable effects on infarct size, cytokine release and cardiac remodeling [38, 69, 72] the competitive P2Y_12_ inhibitor ticagrelor was not used due to known pleiotropic effects beyond P2Y_12_ receptor inhibition, i.e. inhibition of toll like receptors-1/2 (TLR1/2), the protease activated receptor (PAR)-pathway [71] and the equilibrative nucleoside transporter 1 (ENT1), which increases extracellular concentrations of adenosine [2]. Ubiquitous P2Y_12_ knockout or prasugrel treatment both attenuated hematopoietic BM activation after MI, reflected in reduced cell cycle activity of LSK cells, which transferred to lower numbers of downstream progenitors and leukocytes of myeloid and lymphocyte origin in the blood. Intriguingly, neutropenia has been described as an adverse drug reaction in some patients treated with P2Y_12_ inhibitors and, vice versa, a small but significant rise in blood neutrophil counts after terminating P2Y_12_ antagonist therapy has been recorded [62, 75]. Next to myeloid cells, lymphocytes are further key players in the remodeling of cardiac lesion [26, 27]. Hence, P2Y_12_ inhibition seems to not only modulate innate but also adaptive immunity after MI.

Scaled down emergency hematopoiesis and reduced leukocytosis after MI by P2Y_12_ inhibition with prasugrel translated to limited leukocyte infiltration of primarily myeloid origin to the infarct, ameliorated adverse cardiac remodeling and preserved cardiac function after MI. Notably, P2Y_12_ receptor inhibition resulted in higher collagen content in the infarct border zone. While exaggerated post-MI cardiac fibrosis especially outside the infarct zone has been associated with reduced cardiac compliance [66], locally restricted increase of the collagen portion in the border zone may also indicate a well-balanced wound healing with increased tissue stability. Supported by signs of neovascularization, P2Y_12_ inhibition post-MI preserved cardiac function and reduced adverse cardiac remodeling.

Crosstalk between the injured myocardium and hematopoietic BM uses multiple channels, moderated by soluble danger signals such as pro-inflammatory interleukin-1β and the sympathetic nerve system (SNS). [6, 25, 57] BM retention factors, namely CXCL12, VCAM1, SCF and angiopoietin, are secreted by regulatory BM niche cells and modulate LSK homeostasis [42]. Both IL-1β and SNS induce downregulation of BM retention factors to activate LSK cells [10, 57]. Putting our findings in the context of the appreciated signaling pathways that promote emergency hematopoiesis after MI, we evaluated cornerstones of IL-1β-mediated and sympathetic nervous signaling under the treatment with P2Y_12_ antagonist prasugrel. Plasma levels of IL-1β after MI as well as expression of BM niche factors were not affected by P2Y_12_ inhibition with prasugrel. Also, the release of LSK and progenitor cells from the BM into circulation, previously described to play an essential role for IL-1β-dependent extramedullary hematopoiesis after MI [57], remained unchanged by prasugrel treatment and supports the hypothesis of a direct ADP-dependent and P2Y_12_ receptor-mediated effect on LSK cells.

In the setting of ischemic myocardial injury and pressure overload [39, 76], P2Y_12_ receptor inhibition alleviates adverse cardiac remodeling and preserves cardiac function via platelets’ immunoregulation, closely linked to leukocyte recruitment with platelet-leukocyte, platelet-endothelial and enhanced leukocyte-endothelial interactions [16, 39]. In this context, P2Y_12_ inhibition was shown to limit platelet-leukocyte-conjugation by reducing platelet p-selectin expression [39], preventing leukocyte rolling for transendothelial migration [70]. To distinguish our findings from platelet-P2Y_12_, we analyzed emergency hematopoiesis in the acute phase after MI in platelet-specific P2Y_12_-deficient GFP chimera. The UBC-GFP reporter allowed us to rule out residual presence of P2Y_12_-competent platelets in recipient mice. As before, prasugrel treatment still reduced LSK cycling in the bone marrow and leukocyte numbers in circulation and the infarcted myocardium in the acute phase after MI, indicating a significant role of P2Y_12_ signaling in inflammation beyond platelet-induced immunoregulation. Furthermore, acetylsalicylic acid (ASA) which inhibits p-selectin expression on platelets, exerted no additional effect on emergency hematopoiesis [30, 41].

Translational investigation of P2Y_12_ signaling in emergency hematopoiesis after MI is limited by the otherwise widely appreciated benefits of P2Y_12_ inhibitors in cardiovascular disease [68]. Yet, there are intriguing observations in support of enhanced anti-inflammatory properties of P2Y_12_ inhibitors. Adding P2Y_12_ antagonists to ASA, considered as dual antiplatelet therapy (DAPT), proved to be superior to any other combination in stable and unstable coronary disease [37, 68]. This benefit was not limited to the expected prevention of target vessel failure but also reduced reoccurring adverse cardiac events [4].

## Conclusion

In our study, we describe a novel pathway in the crosstalk of injured myocardium and the hematopoietic BM after MI, fostering emergency hematopoiesis via ADP-dependent P2Y_12_ receptor-mediated stimulation of upstream hematopoietic stem and progenitor (LSK) cells. Inhibition of the P2Y_12_ receptor modulated the inflammatory response to injury, preserved cardiac function and prevented adverse cardiac remodeling after MI. Given the high demand and turnover of leukocytes following MI [36], the modulation of emergency hematopoiesis may thus be an intriguing approach to target inflammation at its root, prevent excessive secondary myocardial damage and sustain cardiac function.

## Supplementary Information

Below is the link to the electronic supplementary material.

Supplementary file1 P2Y12 receptor expression on LSK cells evaluated by single cell-RNA-sequencing adopted from Klimmeck et al. 2014 [33] (*n* = 3 per group). Mean ± S.E.M. (PNG 54 KB)

Supplementary file2 Proliferation analysis performed with BrdU incorporation assay in C57BL/6 mice treated with prasugrel compared to vehicle control. Bar graph shows relative cycling LSK rates in % on day 3 after MI. (*n* = 6–7 per group; Mann–Whitney test). Mean ± S.E.M., *p < 0.05. (PNG 132 KB)

Supplementary file3 **A** IL-1β levels in plasma on day 1 after MI in prasugrel-treated C57BL/6 mice compared to vehicle control and wildtype (WT), assessed by ELISA (*n* = 12–15 per group; Kruskal-Wallis test). **B** Bone marrow niche factors CXCL12, VCAM-1, Angpt-1 and SCF on day 3 after MI in C57BL/6 mice under prasugrel treatment compared to vehicle control and WT, evaluated by qPCR (*n* = 6–10 per group; Kruskal–Wallis test). **C** LSK cell and hematopoietic progenitor mobilization into peripheral blood on day 3 after MI under prasugrel treatment in comparison to vehicle control, evaluated by FACS (*n* = 6–8 per group; Mann–Whitney test). Mean ± S.E.M., **p < 0.01. (PNG 222 KB)

Supplementary file4 **A** Schematic illustration of the experimental setup. After establishing cyclooxygenase-mediated platelet inhibition by a loading dose of ASA, LAD was ligated for MI and analysis was performed on day 2 after MI as shown. **B** Bleeding time under oral treatment with ASA versus vehicle control (*n* = 3–4 per group; Mann-Whitney test). **C** Flow cytometric gating for LSK cells and cell cycle analysis performed with Ki67/DAPI assay in ASA-treated C57BL/6 mice compared to vehicle control. Bar graphs show cycling LSK rates and LSK in non G0-phase in % and cell counts of the progenitor populations GMP and MDP per femur on day 2 after MI (*n* = 5–8 per group; Mann–Whitney test). Mean ± S.E.M., *p < 0.05. (PNG 449 KB)
