# Supplementary figures and images for: P2Y12-dependent activation of hematopoietic stem and progenitor cells promotes emergency hematopoiesis after myocardial infarction
Source: Basic Res Cardiol. 2022 Mar 30;117(1):16. doi: 10.1007/s00395-022-00927-6 (PMC8967792; doi:10.1007/s00395-022-00927-6)

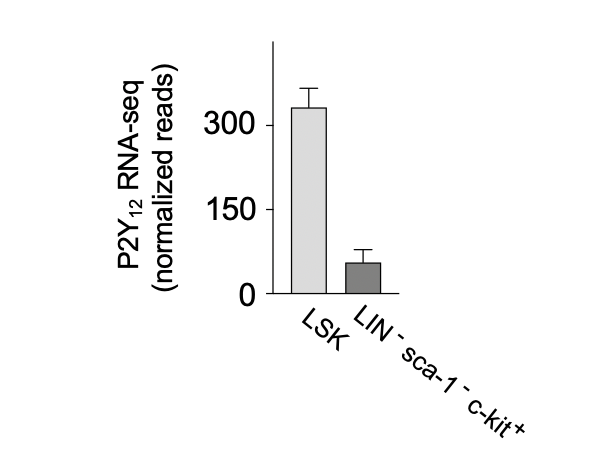

Supplement: Supplementary file 1 — Supplementary file1 P2Y12 receptor expression on LSK cells evaluated by single cell-RNA-sequencing adopted from Klimmeck et al. 2014 [33] (n = 3 per group). Mean ± S.E.M. (PNG 54 KB) [file 395_2022_927_MOESM1_ESM.png]

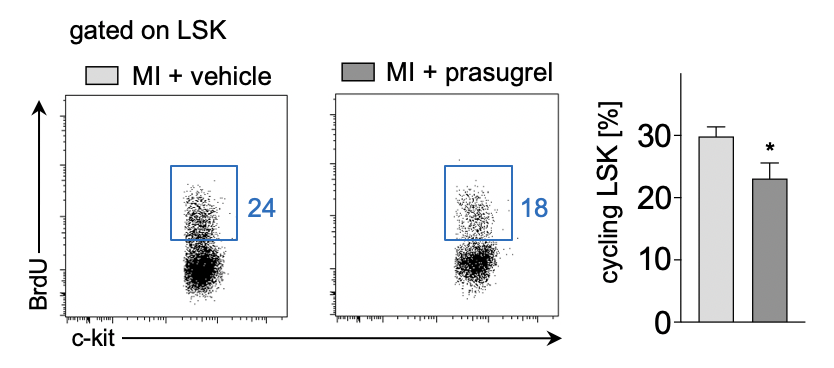

Supplement: Supplementary file 2 — Supplementary file2 Proliferation analysis performed with BrdU incorporation assay in C57BL/6 mice treated with prasugrel compared to vehicle control. Bar graph shows relative cycling LSK rates in % on day 3 after MI. (n = 6–7 per group; Mann–Whitney test). Mean ± S.E.M., *p < 0.05. (PNG 132 KB) [file 395_2022_927_MOESM2_ESM.png]

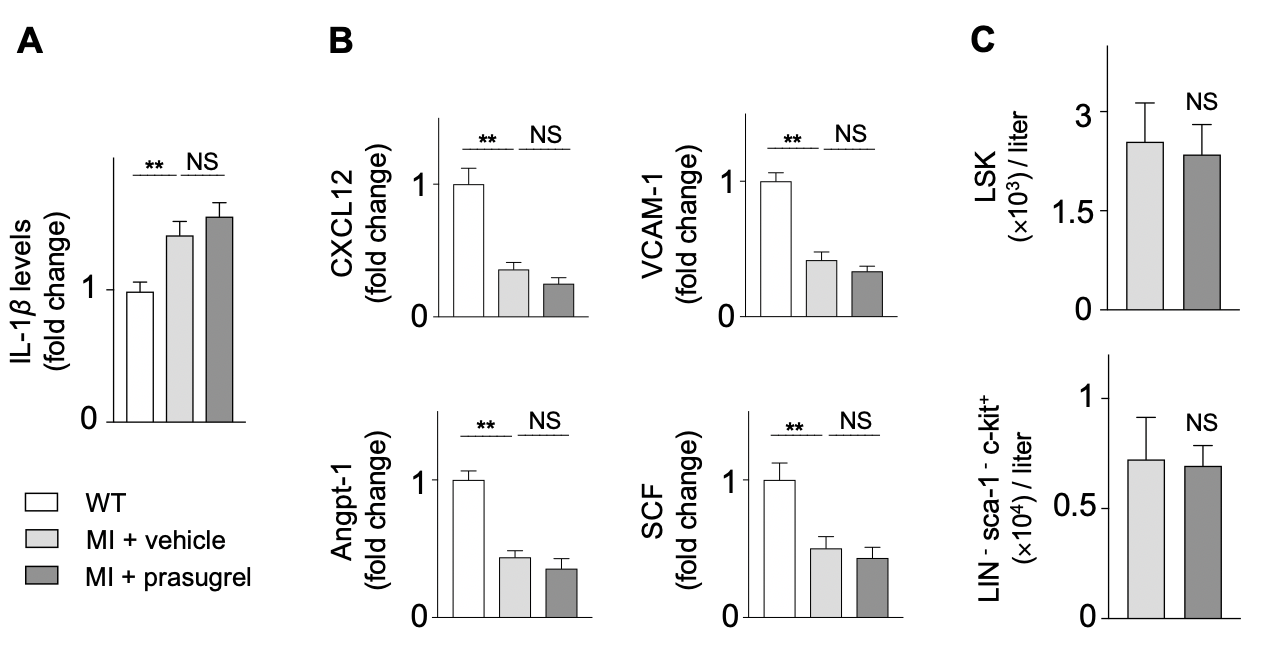

Supplement: Supplementary file 3 — Supplementary file3 A IL-1β levels in plasma on day 1 after MI in prasugrel-treated C57BL/6 mice compared to vehicle control and wildtype (WT), assessed by ELISA (n = 12–15 per group; Kruskal-Wallis test). B Bone marrow niche factors CXCL12, VCAM-1, Angpt-1 and SCF on day 3 after MI in C57BL/6 mice under prasugrel treatment compared to vehicle control and WT, evaluated by qPCR (n = 6–10 per group; Kruskal–Wallis test). C LSK cell and hematopoietic progenitor mobilization into peripheral blood on day 3 after MI under prasugrel treatment in comparison to vehicle control, evaluated by FACS (n = 6–8 per group; Mann–Whitney test). Mean ± S.E.M., **p < 0.01. (PNG 222 KB) [file 395_2022_927_MOESM3_ESM.png]

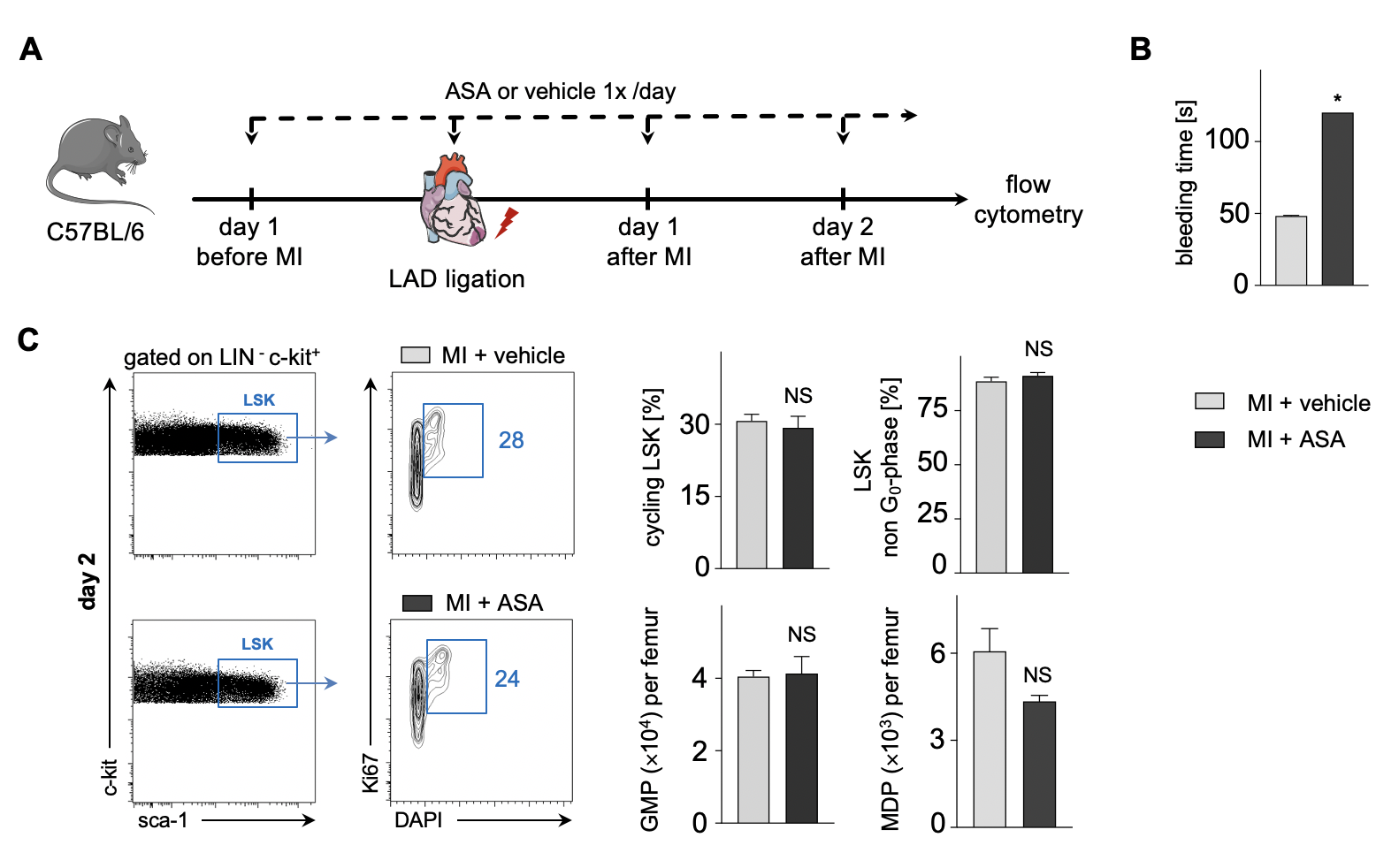

Supplement: Supplementary file 4 — Supplementary file4 A Schematic illustration of the experimental setup. After establishing cyclooxygenase-mediated platelet inhibition by a loading dose of ASA, LAD was ligated for MI and analysis was performed on day 2 after MI as shown. B Bleeding time under oral treatment with ASA versus vehicle control (n = 3–4 per group; Mann-Whitney test). C Flow cytometric gating for LSK cells and cell cycle analysis performed with Ki67/DAPI assay in ASA-treated C57BL/6 mice compared to vehicle control. Bar graphs show cycling LSK rates and LSK in non G0-phase in % and cell counts of the progenitor populations GMP and MDP per femur on day 2 after MI (n = 5–8 per group; Mann–Whitney test). Mean ± S.E.M., *p < 0.05. (PNG 449 KB) [file 395_2022_927_MOESM4_ESM.png]
